# The use of oxygen reserve index in one-lung ventilation and its impact on peripheral oxygen saturation, perfusion index and, pleth variability index

**DOI:** 10.1186/s12871-021-01539-8

**Published:** 2021-12-20

**Authors:** Gonul Sagiroglu, Ayse Baysal, Yekta Altemur Karamustafaoglu

**Affiliations:** 1 Department of Anesthesiology and Reanimation, Trakya University Faculty of Medicine, Edirne, Turkey; 2 Pendik District Hospital, Clinic of Anesthesiology and Reanimation, Pendik, 34980 Istanbul, Turkey; 3 Department of Thoracic Surgery, Trakya University Faculty of Medicine, Edirne, Turkey

**Keywords:** One lung ventilation, Hypoxemia, Oxygen reserve index, Perfusion index, Pleth variability index

## Abstract

**Background:**

Our goal is to investigate the use of the oxygen reserve index (ORi) to detect hypoxemia and its relation with parameters such as; peripheral oxygen saturation, perfusion index (PI), and pleth variability index (PVI) during one-lung ventilation (OLV).

**Methods:**

Fifty patients undergoing general anesthesia and OLV for elective thoracic surgeries were enrolled in an observational cohort study in a tertiary care teaching hospital. All patients required OLV after a left-sided double-lumen tube insertion during intubation. The definition of hypoxemia during OLV is a peripheral oxygen saturation (SpO2) value of less than 95%, while the inspired oxygen fraction (FiO2) is higher than 50% on a pulse oximetry device. ORi, pulse oximetry, PI, and PVI values were measured continuously. Sensitivity, specificity, positive and negative predictive values, likelihood ratios, and accuracy were calculated for ORi values equal to zero in different time points during surgery to predict hypoxemia. At Clinicaltrials.gov registry, the Registration ID is NCT05050552.

**Results:**

Hypoxemia was observed in 19 patients (38%). The accuracy for predicting hypoxemia during anesthesia induction at ORi value equals zero at 5 min after intubation in the supine position (DS5) showed a sensitivity of 92.3% (95% CI 84.9–99.6), specificity of 81.1% (95% CI 70.2–91.9), and an accuracy of 84.0% (95% CI 73.8–94.2). For predicting hypoxemia, ORi equals zero show good sensitivity, specificity, and statistical accuracy values for time points of DS5 until OLV30 where the sensitivity of 43.8%, specificity of 64%, and an accuracy of 56.1% were recorded. ORi and SpO2 correlation was found at DS5, 5 min after lateral position with two-lung ventilation (DL5) and at 10 min after OLV (OLV10) (*p* = 0.044, *p* = 0.039, *p* = 0.011, respectively). Time-dependent correlations also showed that; at a time point of DS5, ORi has a significant negative correlation with PI whereas, no correlations with PVI were noted.

**Conclusions:**

During the use of OLV for thoracic surgeries, from 5 min after intubation (DS5) up to 30 min after the start of OLV, ORi provides valuable information in predicting hypoxemia defined as SpO2 less than 95% on pulse oximeter at FiO2 higher than 50%.

## Introduction

### One-lung ventilation and thoracic surgeries

There is an ongoing investigation to provide advanced monitoring techniques during thoracic surgeries that require one-lung ventilation (OLV). For patients with a possible diagnosis of lung tumor, the surgical team performs either a video-assisted thoracoscopy (VATS) or thoracotomy surgical procedures. The anesthesiologists perform OLV in a lateral decubitus position after a double-lumen tube (DLT) insertion during tracheal intubation. There is usually a request from the surgeon for a collapsed lung where they perform the operative procedure in a surgical field. The lower, dependent lung is ventilated, whereas the upper, non-dependent lung collapses when opening the chest. There is perfusion in this lung, causing a transpulmonary shunt without ventilation. The transpulmonary shunt in the non-dependent lung is the main reason for hypoxemia during OLV. This hypoxemia in the upper deflated lung causes a physiological mechanism called hypoxic pulmonary vasoconstriction (HPV) which is responsible for diverting blood flow from the non-ventilated lung to the ventilated lung. Therefore, HPV causes a decrease in ventilation-perfusion mismatch and improves arterial oxygenation [1–4]. There are other causes of hypoxemia [2, 3, 5]. Despite the correct placement of the DLT, hypoxemia occurs in approximately 10 to 25% of patients and routine use of flexible brochoscopy for positioning of the DLT decreased the incidence of hypoxemia [3, 5].

### Definition of hypoxemia during one-lung ventilation

The definition of hypoxemia during OLV is a peripheral oxygen saturation (SpO_2_) value of less than 95% while the inspired oxygen fraction (FiO_2_) is 50% or higher on a pulse oximetry device [4]. Mild hypoxemia is considered where SpO_2_ values are between 95 and 90% meanwhile, arterial partial pressure of oxygen (PaO_2_) values from arterial blood gas analysis show values of 75–60 mmHg. Severe hypoxemia refers to a SpO_2_ value of less than 90% and corresponds to PaO_2_ values of less than 60 mmHg [3, 4]. A derivative of arterial oxygen saturation can be measured peripherally as SpO_2_ using a non-invasive monitoring device called pulse oximetry. This device measures the level of PaO_2_ in the range of 0 to 100 mmHg where FiO_2_ value is equal to 21%. However, a pulse oximetry device cannot consistently detect desaturation when FiO_2_ is greater than 50% [2, 3, 5].

### Pulse oximetry versus oxygen reserve index for detection of hypoxemia and hyperoxemia

The Oxygen Reserve Index (ORi) is a multiwavelength pulse oximeter, and it provides continuous analysis of PaO_2_ values of moderate hyperoxia at a range of 100–200 mmHg [2–9]. This device can measure several oximeter-related parameters including; ORi, SpO_2_, perfusion index (PI), and perfusion pleth variability (PVI). The multiwave pulse co-oximetry device can provide a calculated ORi for pulse oximetry values greater than 98%. If we could give an example, it would be an incidence where a falling PaO_2_ value approaches 100 mmHg, and a SpO_2_ value is higher than 98%. The multiwave oximeter device measures an ORi value that decreases and approaches a value of 0.24 [9]. This observation in a previous study provided data that ORi may provide information in both clinical situations where there is an impending hypoxic state or an unintended hyperoxic state [6–10]. ORi parameter offers a value that ranges between “1,” which shows a significant oxygen reserve, to “0,” which reveals no oxygen reserve. ORi begins to increase from 0.00 at a PaO_2_ value of 100 mmHg and reaches a plateau of 1.00 at a PaO_2_ value of 200 mmHg.

### Other oximeter parameters: perfusion index (PI), and pleth variability index (PVI)

PI is an indicator of the relative strength of the pulsatile signal from a pulse oximetry device. A higher PI value shows that the pulsatile movement increases, and peripheral circulation at the sensor site improves accordingly. The PVI is a relative variability in the pleth waveform and provides a value between 0 and 100 in a noninvasive measurement from a pulse oximetry device. PVI is an automatic measurement of the dynamic change in PI that occurs during a complete respiratory cycle [11, 12].

### Main objective of the study

The main objective of this study is to investigate the effects of ORi parameter on hemodynamical parameters (heart rate and blood pressure) and oximeter-related parameters such as; peripheral oxygen saturation, PI, and PVI during elective thoracic surgeries requiring OLV and general anesthesia.

## Methods

### Patients and settings

The investigators performed a prospective observational cohort study in 14 months on patients requiring elective thoracic surgery for open lung resection via a thoracotomy or VATS at the Trakya University School of Medicine Hospital, Edirne, Turkey. The investigators conducted the study between 2020 and 2021. After the Hospital Ethics Committee (TÜTF-BAEK 2020/108), the investigators recruited patients for this clinical study. Out of a total of 59 patients, 50 patients with a diagnosis of lung tumor underwent either VATS or open thoracotomy. The surgical procedures during these operations include; either lobectomy, pneumonectomy, lung biopsy, or wedge resection. The Human Research Ethics Committee of Trakya University Medical Faculty, Edirne, Turkey approved this clinical study protocol. The investigators collected written informed consent from patients or their relatives for this clinical study during preoperative visits. The study is registered in the Clinicaltrials.gov registry, and our Registration ID is NCT05050552. The pulmonary function tests, including the percentage of expected, forced expired volume during the first second (FEV_1_%), the ratio of FEV_1_/FVC% (percentage of expected forced vital capacity to FEV_1_) were done in some patients with a possible diagnosis of severe lung disease because of the global pandemia in 2020 and 2021. Patients with FEV_1_ between 30 and 80% and FEV_1_/FVC ratio of < 70% were considered as having a moderate level of chronic obstructive pulmonary disease as per literature. These patients were included whereas, severely restricted patients were excluded [2, 3].

Inclusion criteria included; patients at ages between 22 and 80 years old, American Society of Anesthesiologists Physical Status (ASA-PS) risk groups of 1 to 3, surgical procedures of either open lung resection with thoracotomy or VATS, general anesthesia including sevoflurane inhalational anesthesia during maintanence, the use of DLT and OLV. Exclusion criteria include; refusal to participate in a study, history of severe asthma, preoperative renal insufficiency (creatinine > 114 umol/L); preoperative liver dysfunction (aspartate amino transferase-AST > 40 U/L, alanine amino transferase-ALT > 40 U/L); previous history of coronary or vascular disease or heart failure with an ejection fraction less than 40%, lung function study showing an FEV_1_ less than 50%, history of severe chronic respiratory disease of the non-operated lung, pregnancy, history of previous pulmonary resection and hemoglobinopathies [8, 9, 13].

### The anesthetic management, definition of hypoxemia and collected data during OLV

The investigators did not administer drugs for premedication to prevent hypoxemia-related events. After admitting a patient to the operating theatre, anesthesiologists applied electrocardiogram, noninvasive blood pressure and pulse oximetry monitoring devices, and measured these parameters continuously. The monitored parameters include; heart rate (HR), mean arterial pressure (MAP), systolic blood pressure (SBP), diastolic blood pressure (DBP), and SpO_2_. The anesthesiologists provided general anesthesia using intravenous doses of propofol (Pofol, Fresenius Pharmaceutical, Turkey), 2 to 3 mg/kg, rocuronium (Esmeron, Organon Pharmaceuticals, USA) at a dose of 0.6 mg/kg, and fentanyl (Janssen fentanyl, Janssen Pharmaceutical, Belgium) at a dose of 2 to 3 mcg/kg. The anesthesiologist placed a 20 Gauge radial artery catheter on all patients and connected it to a disposable pressure transducer to provide continuous monitoring following the induction of anesthesia. During tracheal intubation, a left Robertshaw DLT was used. The anesthesiologist used a flexible broncoscopy for correct positioning of DLT in supine and lateral decubitus positioning. For anesthetic maintenance, anesthesiologists used inhalational anesthetic of sevoflurane (Sevorane, Abbott Pharmaceutical, USA) at an end-tidal concentration of 1 to 2% and intravenous fentanyl boluses at a dose of 0.5 to 1 microgram/kg every hour. The hemodynamical stability was maintained during the surgical procedures where keeping HR between 60 and 100 beats/minute and keeping MAP between 60 and 80 mmHg. During surgery, intravenous rocuronium was used every hourly at a dose of 0.05 mg/kg. All patients received an intravenous infusion of lactated Ringer’s solution at a dose of 10 ml/kg/hr.

Hemodynamical and oximeter-related data of HR, MAP, SBP, DBP, SpO_2_, PaO_2_, ORi, PI, and PVI values were recorded at thirteen different time points during anesthesia induction and maintenance of the surgery. Radical-7 Pulse CO-Oximeter is used to measure oximeter parameters of ORi, PI, and PVI (Masimo Inc., Irvine, CA, USA). During the collection of these parameters, the investigators measured peripheral oxygen saturation using a Pulse CO-Oximetry probe. For other oximeter-related parameters, the Rainbow R1 25-L probe was used, a product of the same company [8, 9]. Baseline values of ORi provide data before preoxygenation, and afterward, patients were pre-oxygenated with 100% oxygen. Therefore, the list of time points for collection of data include as follows; first, during the patient’s arrival to the operating room in the supine position breathing room air (basal), during preoxygenation with 100% oxygen in the supine position (preoxygenation), 5 min after tracheal intubation during two-lung ventilation in the supine position (ORiDS5), 5 min after placing the patient in a lateral position with two-lung ventilation (ORiDL5), at 1 min after OLV placement (OROLV1), and afterwards; at 2 min (OROLV120), 5 min (OROLV5), 10 min (OROLV10), 15 min (OROLV15), 30 min (OROLV30), 45 min (OROLV45), 60 min (OROLV60) and 90 min after OLV placement (OROLV90) [8, 9, 13, 14].

After general anesthesia induction and intubation, the anesthesiologists provided mechanical ventilation, and two lung ventilation in the supine position required the settings of a tidal volume of 8–10 mL/kg, inspiration to expiration ratio of 1:2, and respiratory rate of 10–12/min, without positive end-expiratory pressure (PEEP). During operation, the surgical team provided a lateral decubitus position before incision and the anesthesiologist initiated OLV after positioning. The dependent lung was ventilated with a tidal volume of 6–8 mL/kg, I: E ratio of 1:2, respiratory rate of 12–14/min with an unchanged FiO_2_ of 0.5 with an Aestiva 3000 ventilator (Datex-Ohmeda Inc. Madison, U.S.A.) [6, 15]. During surgery, the anesthesiologists were responsible for the anesthesia maintenance with the use of anesthetic agents such as; inhalational anesthesia of sevoflurane, intravenous rocuronium maintenance dose of 0.05 mg/kg every hourly, and intravenous fentanyl maintenance dose of 1 to 2 mcg/kg.

Hypoxemia during OLV is a SpO_2_ value of less than 95% while the FiO_2_ is 50% or greater on a pulse oximetry device [4, 5, 9]. The anesthesiologist who conducts the anesthesia during surgery was responsible for increasing FiO_2_, using bag-mask ventilation of 100% for a while, implementing an alveolar recruitment maneuver, or using continuous positive airway pressure to the collapsed lung during a desaturation of SpO_2_ value less than 95% [2, 3, 8, 9, 11, 13]. A flexible broncoscopy was present during the whole surgical procedure to detect malpositioning of the DLT. The investigators recorded the duration of surgery, anesthesia, and duration of OLV.

### The management of hypoxemic events and other unwanted events during surgery

The anesthesiologists provided oxygen titration depending mainly on the SpO_2_ values in our study group of patients. The data collectors were usual residents in anesthesiology. The residents performed a blood gas analysis at DL5 time point only. The reason for the abscence of this routine arterial blood gas analysis during thoracic surgeries was a recent colloborative decision of our hospital and anesthesiology department to decrease medical costs. In addition, although arterial blood gases analysis is crucial to document the exact measurement of oxygenation via PaO_2_ values, it is impractical to obtain real-time values during an episode of hypoxemia [8, 9].

After induction, patients were routinely ventilated with 50% FiO_2_ (50% oxygen + 50% air mixture, 1 l/minute fresh gas flow). The anesthesiologist was responsible for keeping SpO_2_ values greater than 94. For this purpose, necessary adjustments in FiO_2_ values and mechanical ventilation parameters as well as necessary maneuvers were performed to provide better oxygenation. The incidence of thromboembolic complications, arrhythmias, pneumonia, the duration of hospital and intensive care unit stay were recorded [9, 11, 13–18]. Intravenous ephedrine (Ephedrine, Osel Pharmaceutical, Turkey) at a dose of 10 mg bolus injections were considered if SBP was less than 90 mmHg. Hypotension was defined as a decrease in MAP more significant than 20% after anesthesia induction and treated with intermittent bolus doses of 5 mg ephedrine. The definition of hypotension was based on previous studies [12].

### Summary of surgical procedure

Surgical resection was performed through a posterolateral thoracotomy. A suspicious tumor was located, and if possible all necessary frozen section samples were obtained for pathological evaluation. At the end of the operation, the suspicious mass was removed from its location. The necessary suturing, aspiration, and irrigation of fluids and blood were performed [14, 15, 18].

### The ethical considerations

Trakya University Faculty of Medicine University Ethical Committee agreed and approved the study in February 2020. All patients approved the fully informed written consent to participate in the study. The participants had confidentially during the study process and were able to withdraw from the research process at any time. The investigators discussed any expected benefits or potential harm for the research in detail.

### Statistical analysis

The investigators used an SPSS 15.0 (Statistical Package for Sciences, USA) program to analyze the data of our clinical study. Data were presented as mean ± SD and numbers (percentages), as indicated. Normality was tested with the Kolmogorov-Smirnov test. Some parameters are reported as median (interquartile range [IQR], 25th to 75th percentile). Sensibility, specificity, positive and negative predicted values, likelihood ratios, and their respective confidence intervals were obtained from a two-by-two contingency table for the validity of ORi equals to zero during different moments before and after OLV was achieved to predict the first hypoxemia (SpO_2_ value of < 95%) episode after OLV [8, 9, 13]. The proportion of true positives and true negatives in all evaluated cases was considered to be accurate. The level of statistical significance was a *p*-value of less than 0.05. For calculation of sample size, a hypoxemia rate of 30% after OLV, and a 10% precision at 95% confidence intervals, an alpha error of 0.05, and a power of 80%, the number of patients for the study was calculated as 28 patients [8, 13, 14].

## Results

The investigators performed the clinical study on 50 patients in 14 months duration. The median age of the whole group was 53 years (22–80). There were 28 males and 22 females. The data presented in Table 1 provides demographic information, co-morbidities, pulmonary function tests of 26 patients with possible moderate to severe lung disorders, surgical approach and type of surgery. Pulmonary function tests were not obtained from all patients due to the COVID-19 pandemic. Hemodynamic and oximeter data that are described in methods section were continuously monitored and collected at several phases of anesthesia and surgery. The residents performed arterial blood gas analysis at only one time point which is DL5 time. The residents were responsible to record pulse oximetry and other oximeter values for detection of hypoxemic episodes.

**Table 1:** Demographic data and operation characteristics of undergoing elective thoracic surgery with open lung ventilation

Age, (year)	55.46 ± 13.85
Height, (cm)	168.5 ± 8.43
Weight, (kg)	77.76 ± 16.1
Body mass index, (kg/m2)	27.54 ± 6.17
Gender, n (%)	
Female	22 (44)
Male	28 (56)
ASA-PS, n (%)	
I	5 (10)
II	27,854)
III	18 (36)
FVC, (mL)	2.87 ± 0.68
FEV1	2.3 ± 0.59
Smoking, n (%)	34 (68)
COPD, n (%)	11 (22)
Hypertension, n (%)	17 (34)
Diabetes mellitus, n (%)	8 (16)
Coronary artery disease, n (%)	6 (12)
Right side intervention, n (%)	24 (48)
Surgical approach, n (%)	
Thoracotomy	27 (54)
VATS	23 (46)
Type of surgery, n (%)	
Lung biopsy	12 (24)
Wedge resection	19 (38)
Lobectomy	14 (28)
Pneumonectomy	5 (10)
Duration of operation, (min)	71.3 ± 37.59

*ASA-PS* American Society of Anesthesiologists-physical status, *BMI* Body mass index, *COPD* Chronic obstructive pulmonary disease, *FVC* Forced vital capacity, *FEV*_*1*_ Forced expiratory volume fist second, *VATS* Video assisted; thoracoscopic surgery

Table 2 shows the data analysis of ORi equals to 0 for predicting hypoxemia at different time points during anesthesia induction and maintenance. The accuracy for predicting hypoxemia during anesthesia induction at ORi value equals zero at DS5 showed a sensitivity of 92.3% (95% CI 84.9–99.6), specificity of 81.1% (95% CI 70.2–91.9), and an accuracy of 84.0% (95% CI 73.8–94.2).

**Table 2:** The data analysis of ORi equals to zero and accuracy for predicting hypoxemia during OLV at different time points of surgery

	Sensitivity	Specificity	PPV	NPV	PLHR	NLHR	Accuracy
Preoxygenation (95% CI)	0.15 (0.1–0.3)	91.9 (84.3–99.5)	40 (26.4–53.6)	75.6 (63.6–87.5)	1.9 (1.9–5.7)	0.9 (0.8–1)	72 (59.6–84.4)
ORIDS5 = 0 (95% CI)	92.3 (84.9–99.6)	81.1 (70.2–.91.9)	63.2 (49.8–76.5)	96.8 (91.9–100)	4.9 (1.1–10.9)	0.1 (0.1–0.2)	84 (73.8–94.2)
ORIDL5 = 0 (95% CI)	69.2 (56.4–82)	83.3 (73–93.7)	81.8 (71.1–92.5)	71.4 (58.9–84)	4.2 (1.4–9.7)	0.4 (0.2–0.5)	76 (64.2–87.8)
OROLV1 = 0 (95% CI)	63.6 (50.3–77)	75 (63–87)	66.7 (53.6–79.7)	72.4 (60–84.8)	2.6 (1.8–6.9)	0.5 (0.3–0.6)	70 (57.3–82.7)
OROLV2 = 0 (95% CI)	65.2 (52–78.4)	70.4 (57.7–83)	68.2 (55.3–81.1)	70.4 (57.7–83)	2.2 (1.9–6.2)	0.5 (0.4–0.6)	69.4 (56.6–82.2)
OROLV5 = 0 (95% CI)	56.5 (42.8–70.3)	66.7 (53.6–79.7)	59.1 (45.5–72.7)	64.3 (51–77.6)	1.7 (0.7–2.7)	0.7 (0.5–0.8)	62 (48.5–75.5)
OROLV10 = 0 (95% CI)	56 (42.2–70)	64 (50.7–77.3)	60.9 (47.3–74.4)	59.3 (50–72.9)	1.6 (0.6–2.6)	0.7 (0.6–0.8)	60 (46.4–73.6)
OROLV15 = 0 (95% CI)	52.2 (38–66.3)	68 (54.8–81.2)	60 (46.1–73.9)	60.7 (46.9–74.5)	1.6 (0.6–2.7)	0.7 (0.6–0.8)	60.4 (46.6–74.3)
OROLV30 = 0 (95% CI)	43.8 (29.7–57.8)	64 (50.4–77.6)	43.8 (29.7–57.8)	64 (50.4–77.6)	1.2 (0.2–2.2)	0.9 (0.8–1)	56.1 (42.1–70.1)
OROLV45 = 0 (95% CI)	40 (23.3–56.7)	72.2 (57–87.5)	54.5 (37.6–71.5)	59.1 (42.3–75.9)	1.4 (0.3–2.7)	0.8 (0.7–1)	57.6 (40.7–74.4)
OROLV60 = 0 (95% CI)	53.3 (35.8–70.9)	68.8 (52.4–85.1)	61.5 (44.4–78.7)	61.1 (43.9–78.3)	1.7 (0.4–3)	0.7 (0.5–0.8)	61.3 (44.1–78.4)
OROLV90 = 0 (95% CI)	50 (25.5–75)	66.7 (43.6–90)	71.4 (49.3–93.6)	44.4 (20.1–68.8)	1.5 (0.2–4.3)	0.8 (0.5–0.9)	56.3 (31.9–80.6)

*ORi* Oxygen reserve index, *OR* Oxygen reserve, *OLV* One-lung ventilation, *PPV* Positive predictive value, *NPV* Negative predictive value, *PLHR* Positive likelihood ratio, *NLHR* Negative likelihood ratio, *CI* Confidental interval, *ORiDS5* ORi under mechanical ventilation 5 min after intubation in supine position, *ORiDL5* ORi under mechanical ventilation 5 min after positioning in the lateral decubitus position, *OROLV1* ORi after 1 min of OLV, *OROLV2* ORi after 2 min of OLV, *OROLV5* ORi after 5 min of OLV, *OROLV10* ORi after 10 min of OLV, *OROLV15* ORi after 15 min of OLV, *OROLV30* ORi after 30 min of OLV, *OROLV45* ORi after 45 min of OLV, *OROLV60* ORi after 60 min of OLV, *OROLV90* ORi after 90 min of OLV

The accuracy for predicting hypoxemia during anesthesia induction at ORi equals zero at 5 min after placing the patient in a ORiDL5 showed a sensitivity of 69.2%, specificity of 83.3%, and an accuracy of 76.0%. The 95% confidence interval (CI) values are presented in Table 2. In this table, the data analysis shows that; for predicting hypoxemia, ORi equals to zero show good sensitivity, specificity and accuracy statistical values for time points of DS5 until OLV30 where sensitivity of 43.8%, specificity of 64%, and an accuracy of 56.1% were recorded. These findings correlated to the previous reports that HPV increases and intrapulmonary shunting decreases after the start of OLV within 30 to 60 min [4, 8, 13, 14].

Overall, from a total of 50 patients in the study group, 19 patients (38%) developed hypoxemia defined as SpO_2_ values of less than 95% at or higher than FiO_2_ value of 50% during the surgical procedure. At the time point of DS5, ORi equals to 0 value was observed in 12 of the 19 patients (63.16%) who presented with hypoxemia. At other time points this hypoxemia was observed as follows; DL5; 11 patients (22%), OLV1; 8 patients (16%), OLV2; 9 patients (18%), OLV5 12 patients (24%) and OLV10 15 patients (30%).

**Fig. 1:**
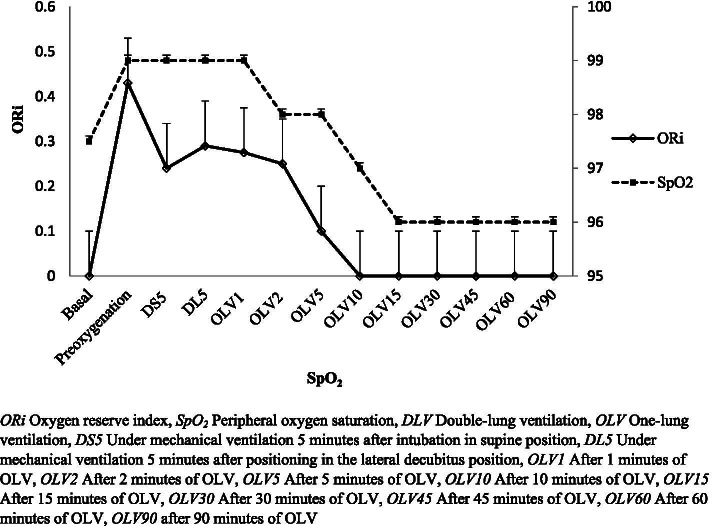
The representative trends of oxygen reserve index (ORi) and peripheral oxygen saturation (SpO_2_) values at different time points during surgery

In Fig. 1, data analysis provides representative trends of ORi and SpO_2_ values in a continuous graph at thirteen different time points during the anesthesia induction and maintenance of the surgery. This correlation showed that; a strong correlation between ORi and SpO_2_ was found at time points of DS5 (*r* = 0.286, *p* = 0.044), DL5 (*r* = 0.293, *p* = 0.039), and, at OLV10 (*r* = 0.360, *p* = 0.011). Therefore, Fig. 1 also supports the relationship between SpO_2_ values and ORi equals to zero values for predicting hypoxemia during anesthesia induction and maintanence.

**Fig. 2:**
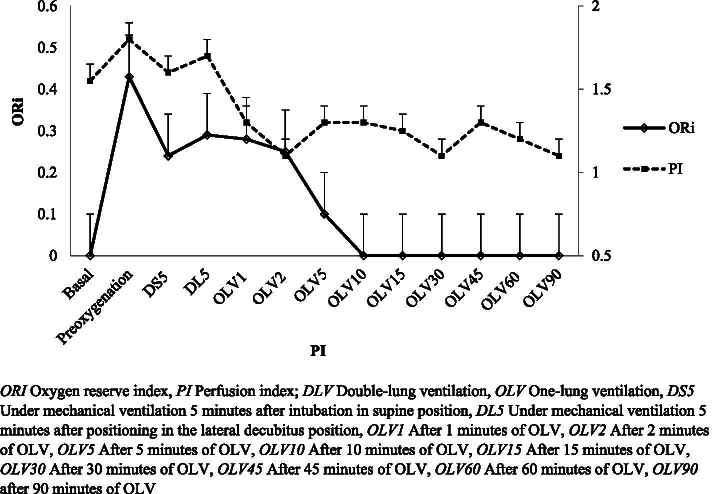
The oxygen reserve index (ORi) and perfusion index (PI) values at different time points of surgery

**Fig. 3:**
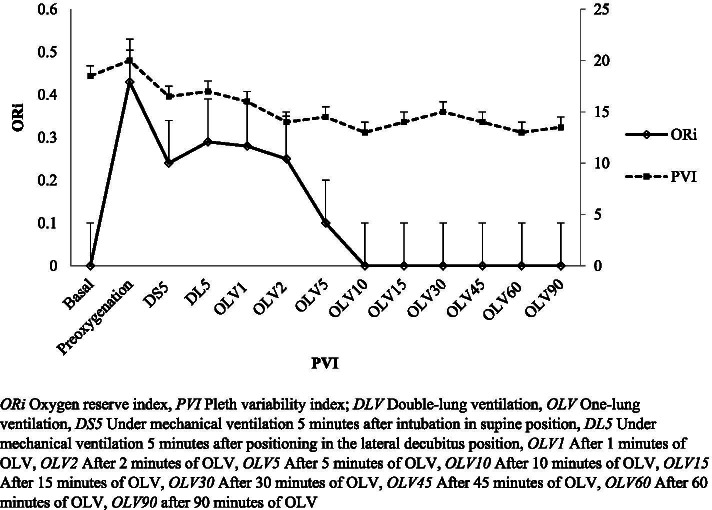
The oxygen reserve index (ORi) and pleth variability index (PVI) values at different time points of surgery

Later, we evaluated the representative trends of the ORi and PI values and the ORi and PVI values at different time points during anesthesia induction and maintenance of thoracic surgeries. These are represented in Figs. 2 and 3.

For hemodynamical and oximeter parameters including; HR, MAP, SBP, DBP, SpO_2_ values, a correlation between these parameters were not found in the statistical analysis (*p* > 0.05). In our study, we demonstrated a time-dependent correlation between PVI and MAP at the time point of OLV90, indicating that PVI showed a relation to MAP at a late stage of the thoracic surgical procedure.

In our study, we investigated the ORi and PVI values at different time points during anesthesia induction and maintenance of thoracic surgery and our findings show that fluid deficit or fluid overload causes changes in PI and PVI values. This is observed in our representative trend graphs in Figs. 2 and 3. Our study provides valuable data for the investigation of correlations between ORi and PI, and PVI. Our study provides data that at a time point of DS5, there is a significant negative correlation with PI (*r* = − 0.332, *p* = 0.019), whereas; no correlations with PVI were noted.

Table 3 shows the median values and interquartile range of PI and PVI values at different measurement points during the study. The analysis of correlations between these PI and PVI values showed a correlation between PI and PVI values at the time point of ORiDL5 (*r* = − 0.284, *p* = 0.046). In other time points, correlations were not demonstrated (*p* > 0.05).

**Table 3:** The median values and interquartile range of perfusion index (PI) and pleth variability index (PVI) values at different measurement points of surgery

Time (min)	Perfusion Index (PI)		Pleth Variability Index (PVI)	
	Median	Interquartile range (IQR)	Median	Interquartile range (IQR)
Baseline	1.55	0.86–2.3	20.5	14–30.25
Preoxygenation	1.8	1.3–2.6	18.5	13–30.25
DS5	1.6	1–2.5	16	11–21
DL5	1.7	1.28–2.3	17	12–26
OLV1	1.3	0.61–1.3	16.5	11.75–23
OLV2	1.1	0.63–1.93	13.5	10–21.25
OLV5	1.3	0.64–1.93	14	10–20.25
OLV10	1.3	0.71–1.7	17	10.5–22.5
OLV15	1.25	0.76–2.1	15	10.25–21
OLV30	1.1	0.66–2	17	10–22
OLV45	1.3	0.82–2.1	14	8.5–20.5
OLV60	1.2	0.63–2.2	14	10–22
OLV90	1.1	0.73–2	13	8.5–18.75

*PI* Perfusion index, *PVI* Pleth variability index, *IQR* Interquartile range, *DLV* Double-lung ventilation, *OLV* One-lung ventilation, *DS5* Under mechanical ventilation 5 min after intubation in supine position, *DL5* Under mechanical ventilation 5 min after positioning in the lateral decubitus position, *OLV1* After 1 min of OLV, *OLV2* After 2 min of OLV, *OLV5* After 5 min of OLV, *OLV10* After 10 min of OLV, *OLV15* After 15 min of OLV, *OLV30* After 30 min of OLV, *OLV45* After 45 min of OLV, *OLV60* After 60 min of OLV, *OLV90* after 90 min of OLV

Table 4 provides time-dependent correlations between ORi with SpO_2_, PI, and PVI. These correlation analysis provide data that ORi has significant correlations with SpO_2_, PI and PVI at some specific time points and these include; at time point of DS5; (*r* = 0.286, *p* = 0.044), DL5 (*r* = 0.293, *p* = 0.039), and OLV10; ORi has a significant correlation with SpO_2_ (*r* = 0.360, *p* = 0.011), at time point of DLS5; ORi has a significant negative correlation with PI (*r* = − 0.332, *p* = 0.019), whereas; 3- no correlations with PVI was noted.

**Table 4:** Time-dependent correlations between oxygen reserve index (ORi) with peripheral oxygen saturation (SpO_2_), perfusion index (PI) and pleth variability index (PVI) during surgery

Time (min)	Peripheral Oxygen Saturation (SpO_**2**_)		Perfusion Index (PI)		Pleth Variability Index (PVI)	
	***r***	***p***	***r***	***P***	***r***	***p***
Preoxygenation	0.121	0.404	0.042	0.774	0.017	0.908
DS5	0.286	0.044*	−0.332	0.019*	0.073	0.617
DL5	0.293	0.039*	−0.010	0.947	0.089	0.540
OLV1	−0.030	0.834	0.020	0.888	−0.013	0.984
OLV2	−0.087	0.548	0.158	0.272	−0.147	0.307
OLV5	−0.249	0.081	0.133	0.358	−0.001	0.997
OLV10	0.360	0.011*	−0.240	0.097	−0.058	0.692
OLV15	0.241	0.099	−0,247	0.091	−0.175	0.234
OLV30	−0.162	0.313	0.305	0.053	−0.189	0.237
OLV45	0.270	0.129	−0.115	0.529	0.038	0.837
OLV60	0.092	0.630	−0.179	0.344	−0.036	0.850
OLV90	−0,412	0.113	0.433	0.094	−0.167	0.535

^*^A *p*-value of less than 0.05 is considered statistically significant.

*ORi* Oxygen reserve index, *SpO*_*2*_ Peripheral oxygen saturation, *PI* Perfusion index, *PVI* Pleth variability index, *DLV* Double-lung ventilation, *OLV* One-lung ventilation, *DS5* Under mechanical ventilation 5 min after intubation in supine position, *DL5* Under mechanical ventilation 5 min after positioning in the lateral decubitus position, *OLV1* After 1 min of OLV, *OLV2* After 2 min of OLV, *OLV5* After 5 min of OLV, *OLV10* After 10 min of OLV, *OLV15* After 15 min of OLV, *OLV30* After 30 min of OLV, *OLV45* After 45 min of OLV, *OLV60* After 60 min of OLV, *OLV90* after 90 min of OLV

## Discussion

The main findings of this study are provided below:

The main conclusion is that ORi is sensitive and specific in predicting hypoxemia defined as SpO_2_ values of less than 95% while the FiO_2_ is 50% or higher on a pulse oximetry device at 5 min after intubation in the supine position (sensitivity of 92.3%, specificity of 81.1% and, an accuracy of 84.0%) [7–9, 13, 15, 17–21].

There are other time points where there is statistically good report of sensitivity, specificity and accuracy for time points at ORiDL5, and during OLV until OLV30 where sensitivity of 43.8%, specificity of 64%, and an accuracy of 56.1% are recorded. These findings correlated to the previous reports that HPV increases and intrapulmonary shunting decreases after the start of OLV within 30 to 60 min [4, 8, 13, 14].

In our study group of patients, a total of 19 patients (38%) developed hypoxemia at various recorded time points during the surgical procedure. ORi provides information for impending hypoxemia that a change in ORi value can be detected 5 to 6 min earlier than pulse oximetry value. Therefore, ORi can provide a valuable time to the anesthesiologist to provide an increase in FiO_2_ values, to perform necessary mechanical ventilation adjustments, to perform aspiration or other anesthetic management techniques to prevent hypoxemia [7–9, 13, 15, 17–21].

During OLV, hypoxemia can develop not only by the intrapulmonary shunt in the non-ventilated lung but also by the ventilation-perfusion mismatch in the ventilated lung or hemodynamic instability [4, 5]. In our study, patients with coronary artery disease and an ejection fraction below 40% were not included into the study. Patients with heart failure were also excluded. During OLV, atelectasis occurs during general anesthesia induction, which causes ventilation/perfusion mismatch even before switching to OLV [5, 6, 10]. During OLV, oxygen delivery to the patient under general anesthesia occurs during various interactions between hemoglobin, oxygen saturation, cardiac output, and normal physiological mechanisms such as HPV and intrapulmonary shunts [3, 4]. Although the causes of OLV-induced hypoxemia are multifactorial, early detection of hypoxemia before the onset of OLV allows the application of different ventilation strategies to improve oxygenation [3–6]. The role of HPV and intrapulmonary shunting are also discussed earlier [4, 10, 14, 22].

A significant correlation between ORi and SpO_2_ was found at time points of DS5, DL5 and, at OLV10. The relationship between SpO_2_ values and ORi equals to zero values for predicting hypoxemia during anesthesia induction and maintenance is supported by these statistical findings. There are previous studies that support these correlations [7–9, 13, 15, 17–21]. In our study group, hypoxemia episodes were observed at various time points throughout the surgery however, the reports were not able to demonstrate a fall of pulse oximeter values below 95% as FiO_2_ values were set at 50% and may have been rised up to 70% after anesthesia management throughout the surgical procedures. In addition to temporary rises in FiO_2_ throughout surgery, mechanical ventilation and anesthetic maneuvers were performed by the anesthesiologists. Because of these interventions, in our opinion, we were not able to show a continuous a correlation between ORi and SpO_2_ values at all measured time points. When ORi which is an oximeter-related parameter is used along with the pulse oximeter monitoring, ORi values may present and record early signs of the downward trend of PaO_2_ in comparison to a pulse oximetry value. In a previous study, at 1 min after start of OLV the measurements show that; hypoxemia was 27.5% where SpO_2_ value was less than 90% whereas; a negative predictive value was reported as 12.9% in those patients who did not achieve an ORi value of 0 at 1 min after the lung collapsed. It has been reported that median time until desaturation was approximately 5.5 to 6 min. Therefore, FiO_2_ values should be kept between 50 to 60% to avoid hyperoxemia and its related adverse effects such as atelectasis [7–9, 13, 17, 18, 20, 21].

Our findings show similarity with a recent study by Alday and his colleagues [8] however, they also suggested that these values may be used to prevent unnecessary hyperoxemia. In our study, it is clear that during anesthetic management FiO_2_ values are kept at a value of 50 to 70% in our patients whereas other studies investigated the use of ORi for hyperoxemia as well [7–9, 13, 17, 18, 20, 21]. In a study by Applegate and his colleagues, a positive correlation between ORi values and PaO_2_ values of 240 mmHg or lower (*r* = 0.536, *p* < 0.01) in comparison to ORi values and PaO_2_ values of higher than 240 mmHg (*r* = 0.0016, *p* > 0.05) [9]. In our study, we were not able to measure PaO_2_ values on each time point because of hospital policies to decrease medical costs. In our study, at the measurement time of arterial blood gas analysis at DL5, we found that 4 patients had a PaO_2_ value above 240 mmHg and ORi values showed statistically significant negative correlation (*r* = − 1.0, *p* < 0.001). In another study, 15 patients undergoing elective thoracic surgery using OLV were evaluated for correlation between PaO_2_ and ORi parameters throughout the surgical procedure and showed that ORi has a significant correlation with PaO_2_ (*r* = 0.671, *p* < 0.001) [18]. There are a few studies that provide evidence that PaO_2_ values show positive correlation with ORi values [7, 9, 11, 18, 20, 21].

During pulse oximetry monitoring, there is a sigmoidal relationship between arterial oxygenation in blood gas value and peripheral oxygenation reported as SpO_2_ value on the pulse oximetry device. This relationship causes no change in pulse oximeter values until PaO_2_ falls below 80 mmHg. Afterward, there is a sudden drop in pulse oximetry value; however, the PaO_2_ is unacceptable for more than 3 to 5 min. Therefore, there is a need to investigate a larger scale of several wavelengths to detect quantitative measurement of methemoglobin, carboxyhemoglobin, and total hemoglobin, and a newly presented device achieved this. Masimo Rainbow Signal Extraction Technology introduced the device [14–16, 19]. ORi is a parameter-driven from this device that is between 0 and 1 values, and it is sensitive to the changes in arterial oxygenation in the blood, with the range of 100 to 200 mmHg [2, 7–9, 13, 15, 18, 20, 21]. When oxygenation is in the moderate hyperoxic content showing an arterial blood oxygenation value of 100–240 mmHg in arterial blood gas analysis, the pulse oximeter SpO_2_ value remains 100%, whereas, there is a decrease in the value of ORi [2, 7–9, 13, 18, 20, 21]. In our study, Fig. 1 and Table 4 provides data on time-dependent correlations between ORi with SpO_2_.

Increased intrathoracic pressure with respiration leads to more immediate reductions in peripheral perfusion in patients with a fluid deficit. In this case, a decrease in the PI value of the patient is observed. As a result of these changes with respiration, the highest and lowest PI ratio corresponds to the PVI. High PVI values are observed in patients with a high fluid deficit or those who do not respond to fluid application changes with changes in the PI [11, 12, 15–17, 23, 24]. In our study, we investigated the ORi and PVI values at different time points during anesthesia induction and maintenance of thoracic surgery and our findings are in correspondence with the previous findings that; fluid deficit or fluid overload causes changes in PI and PVI values. This can be observed in our representative trend graphs in Figs. 2 and 3 [16–18, 23, 24].

Our study provides valuable data for the investigation of correlations between ORi and PI, and PVI. OLV with DLT has significant cardiopulmonary physiological changes, as has been discussed elsewhere [14, 16, 17, 19]. Our study provides data that at a time point of DS5, there is a significant negative correlation with PI (*r* = − 0.332, *p* = 0.019), whereas; no correlations with PVI were noted. This finding is thought to result from anesthesia drugs that are use during anesthesia induction and especially the use of opioid medications [3–6, 10, 12].

The use of FiO_2_ values higher than 50% during anesthesia is related to hyperoxemia, and this high oxygenation decreases cardiac output by reducing heart rate and causing systemic vasoconstriction. Furthermore, hyperoxemia is a potent vasoconstrictor stimulus to the coronary circulation, functioning at the level of the microvascular resistance vessels [7, 21]. Tsuchiya et al. demonstrated that the PVI could be used to evaluate hypotension that is caused secondary to anestethic drugs in patients undergoing general anesthesia without age group classification [23]. This technique has been used in patients undergoing mechanical ventilation in the intensive care unit to detect fluid responsiveness through respiratory patterns and peripheral perfusion changes [11]. There are insufficient data to distinguish the cause of hypotension due to peripheral vasodilatation and fluid redistribution or cardiac output decrease after general anesthesia [23, 24]. High PVI values are observed in patients with a high fluid deficit or those who do not respond to fluid application changes with changes in the PI [24].

In our study, we demonstrated a time-dependent correlation between PVI and MAP at the time point of OLV90, indicating that PVI showed a relation to MAP at a late stage of the surgical procedure. Recently, it is pointed out in a meta-analysis that PVI is a reliable marker in evaluating a response to fluid management [16].

## Limitations

Malpositioning of DLT may cause hypoxemia and, in our study protocol, we included these patients and therefore, this is a limitation of our study [8, 9, 13, 25]. Previously, the arterial blood gas oxygenation results show that PaO_2_ values were higher during right-sided OLV than left-sided OLV. Although it could be predicted that ORi would decrease before the decrease in SpO_2_ during left-sided OLV, the actual extent of this application of either right-sided or left-sided OLV needs to be further evaluated [7, 16, 18, 20, 21]. When oxygenation is in the moderate hyperoxic range of PaO_2_ values between 100 and 240 mmHg, ORi decreases, but SpO_2_ does not [21]. Therefore, ORi values can be used for detection of hyperoxemia however, as we were not able to measure PaO_2_ values secondary to hospital protocol to decrease medical costs, we were not able to evaluate these findings.

## Conclusions

During use of OLV for thoracic surgeries, from 5 min after intubation (DS5) up to 30 min after start of OLV, ORi provides valuable information in predicting hypoxemia defined as SpO_2_ less than 95% on pulse oximeter at FiO_2_ higher than 50%. These findings correlated to the previous reports that HPV increases and intrapulmonary shunting decreases after the start of OLV within 30 to 60 min. ORi provides information for impending hypoxemia that a change in ORi value can be detected 5 to 6 min earlier than pulse oximetry value. Therefore, ORi can provide a valuable time to the anesthesiologist to provide an increase in FiO_2_ values, to perform necessary mechanical ventilation adjustments, to perform aspiration or other anesthetic management techniques to prevent hypoxemia. Fluid responses and anesthesia induction medications has influence over changes in PI and PVI oximeter values. The use of ORi for hyperoxemia during OLV and thoracic surgeries may be useful however, it is not practical as the PaO_2_ values of these patients usually range between 60 mmHg and 200 mmHg and patients are not under risk of hyperoxemia related problems when compared to a higher possible risk of hypoxemia.

## Data Availability

The data is available by permission from Dr. Gonul Sagiroglu, Trakya University, School of Medicine, Depertment of Anesthesiology, Edirne, Istanbul, email address: gonul.sagiroglu45@gmail.com. Please contact this address for permission. Administrative permissions are not required to access the raw data. The authors have agreed to give permission to the data and materials during registration at clinicaltrials.gov.

## Abbreviations

ASA-PS: American Society of Anesthesiologists Physical Status
CI: Confidental interval
DBP: Diastolic blood pressure
DLT: Double lumen tube
FiO_2_: Fraction of inspired oxygen
IQR: Interquartile range
ORi: Oxygen reserve index
OLV: One-lung ventilation
PaCO_2_: Arterial partial pressure of carbon dioxide
PaO_2_: Arterial partial pressure of oxygen
PI: Perfusion index
PVI: Pleth variability index
SpO_2_: Peripheral oxygen saturation
VATS: Video-assisted thoracoscopy
MAP: Mean arterial pressure
SBP: Systolic blood pressure
SD: Standard deviation
